# Best practices in the measurement of circularly polarised photodetectors†‡

**DOI:** 10.1039/d2tc01224c

**Published:** 2022-06-30

**Authors:** Matthew D. Ward, Wenda Shi, Nicola Gasparini, Jenny Nelson, Jessica Wade, Matthew J. Fuchter

**Affiliations:** Department of Physics, Imperial College London South Kensington Campus London SW7 2AZ UK; Centre for Processable Electronics, Imperial College London South Kensington Campus London SW7 2AZ UK jessica.wade@imperial.ac.uk m.fuchter@imperial.ac.uk; Department of Chemistry and Molecular Sciences Research Hub, Imperial College London White City Campus, 82 Wood Lane London W12 0BZ UK; Department of Materials, Imperial College London South Kensington Campus London SW7 2AZ UK

## Abstract

Circularly polarised light will revolutionise emerging technologies, including encrypted light-based communications, quantum computing, bioimaging and multi-channel data processing. In order to make use of these remarkable opportunities, high performance photodetectors that can accurately differentiate between left- and right-handed circularly polarised light are desperately needed. Whilst this potential has resulted in considerable research interest in chiral materials and circularly polarised photodetecting devices, their translation into real-world technologies is limited by non-standardised reporting and testing protocols. This mini-review provides an accessible introduction into the working principles of circularly polarised photodetectors and a comprehensive overview of the performance metrics of state-of-the-art devices. We propose a rigorous device characterisation procedure that will allow for standardised evaluation of novel devices, which we hope will accelerate research and investment in this area.

## Abstract 2

Here we provide a framework of standardised apparatus and tests which enable rigorous characterisation of the performance of photodetectors sensitive to circular polarisation, enabling meaningful comparisons between devices reported in literature.

## Graphical abstract

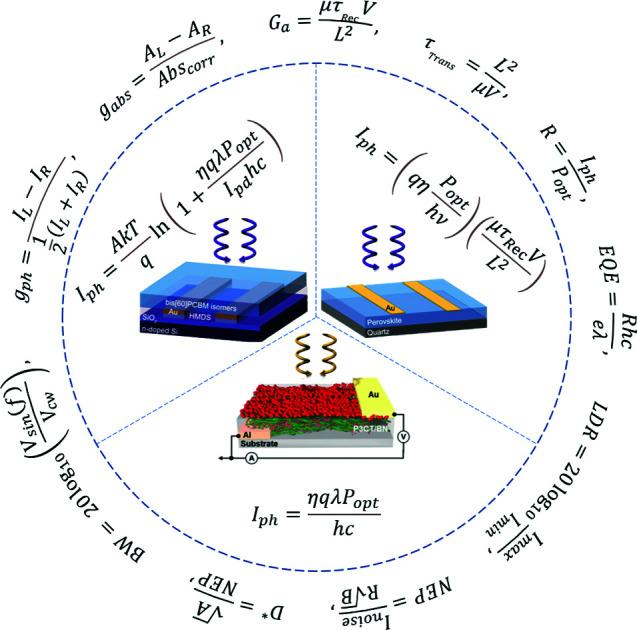

## 1. Introduction

The detection of circularly polarised (CP) light will transform future technologies, including encrypted light-based communications^1,2^ and multi-channel data processing.^3^ The differentiation of left- and right-handed light can facilitate the rapid determination of the absolute configuration of chiral molecules,^4^ polarisation enhanced environmental monitoring through next-generation machine vision^5–7^ and the detection of chiral biomarkers in medical imaging.^8^ Commercially available devices capable of discriminating between left- and right-handed light combine a polarisation insensitive inorganic photodetector with rigid, bulky optical components, which limits their spectral range and makes them both expensive and difficult to miniaturise.

Owing to their tuneable detection wavelengths, simple processing requirements and compatibility with low-cost manufacturing processes, photodetectors based on organic and hybrid organic–inorganic semiconductors have attracted intense research interest in recent years.^9–11^ A variety of these semiconducting materials have been explored for use in such devices, including polymers, small molecules, fullerenes, non-fullerene acceptors and perovskites. Organic based photodetectors are commonly referred to as OPDs; whilst we will use this term here, we note that most of the principles discussed and some of the examples provided involve hybrid or perovskite-based devices. Several OPD device architectures have been considered and strategies to improve device performance (*e.g.* multi-terminal devices, rational material design and modification of charge injection or extraction interfaces) are emerging.^12^ The circularly selective optical response of chiral functional materials makes them an exciting prospect for direct detection of CP light without the need for external optics.^13^ To this end, a variety of organic and organic–inorganic chiral materials have been developed.^14,15^ Due to the strong focus of recent studies on maximising the CP selectivity of detectors, conventional non-CP figures of merit have been largely sidelined. This has led to poor overall performances of devices reported to date, limiting their suitability for widespread application of CP OPDs in real-world technologies. Furthermore, the rapidly growing highly interdisciplinary nature of the research field has resulted in a variety of characterisation protocols, and incomplete reporting of figures of merit, rendering meaningful comparisons between different materials and device architectures a challenge. The development and use of common standards, which were essential for the commercialisation of photovoltaics, photodetectors and light emitting diodes, are desperately needed.

Here we consider the experimental measurements required to characterise CP OPDs rigorously and accurately. We propose a standardised means of reporting their key figures of merit, including Linear Dynamic Range (LDR), Bandwidth and Responsivity. As recent reviews have focussed on the design of materials capable of detecting circularly polarised light and the characterisation of their chiroptical response, we do not provide a further comprehensive review of those aspects here. Instead, we direct readers to several recent and detailed review articles.^13–16^ Here we carefully set out the performance metrics and instrumentation essential to characterise device performance and CP selectivity of OPDs based on chiral functional materials. We hope that this tutorial review serves to advance the field, such that CP OPDs with device performances and sensitivities compatible with the demands of real-world sensing can be realised.

We first provide a brief introduction to the materials systems of current state-of-the-art CP OPDs. We then describe the operating principles of the three most common photodetectors (photodiodes, photoconductors, and phototransistors) and outline the standardised figures of merit of these devices. We conclude by outlining strategies to make testing protocols more robust, which should result in standardised evaluation and reporting of device performance.

## 2. Organic and hybrid organic–inorganic semiconductors for photodetection

There are several materials characteristics which are desirable in the pursuit of high-performance photodetectors, including large optical absorption coefficients and long recombination times. Advances in molecular design have improved the spectral sensitivity of and charge transport in non-chiral organic and hybrid organic–inorganic materials, although some of these strategies do not transfer cleanly to chiral materials due to inherent differences in molecular shape, packing, *etc.*

Organic semiconductors display much lower dielectric constants than their inorganic counterparts (*ε*_r_ ∼ 3.5 *vs. ε*_r_ ≥ 10), which results in the formation of Coulombically bound electron–hole pairs (excitons) under optical excitation. As thermal dissociation is highly improbable in a low dielectric constant medium, the generation of free charges in organic semiconductors typically requires the intermixing of two semiconductors with suitable energetics to yield a heterojunction between an electron donating and an electron accepting component.^17^ The electron accepting or electron donating character of the organic semiconductor is controlled through chemical design, for example by incorporating electron withdrawing or donating groups into the molecular structure.

**Fig. 1:**
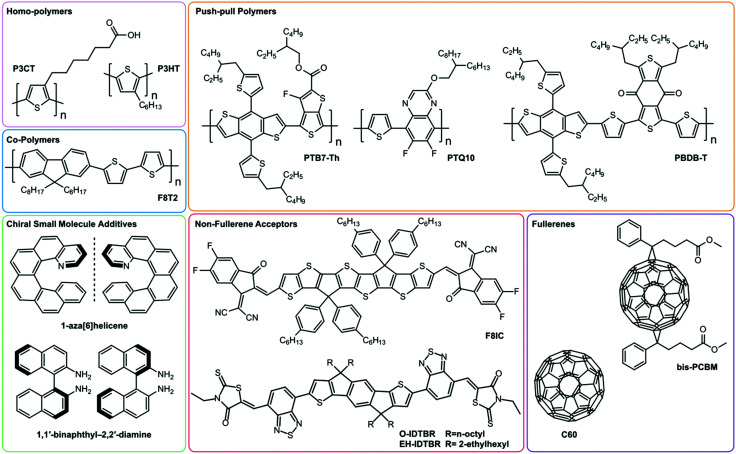
Summary of the π-conjugated organic semiconducting molecules discussed as part of this review, grouped into homo-polymers, co-polymers, chiral small molecule additives, push-pull polymers, non-fullerene acceptors and fullerenes.

Until recently, the most common selection of non-chiral materials in OPDs comprised homopolymer donors, such as P3HT, or push–pull polymers (PTB7-Th, PBDB-T, PTQ10), in combination with fullerene-based acceptors (see Fig. 1).^18,19^ Recently, non-fullerene acceptors (NFAs), such as IDTBR, have emerged as alternatives to fullerene-based acceptors with broader spectral photosensitivity and improved long-term morphological stability whilst retaining good charge-transport properties.^18,19^ Most NFAs make use of push-pull hybridisation, which allows them to absorb strongly in the visible and NIR region of the solar spectrum, achieving higher voltage outputs compared to fullerene-based alternatives.^18,19^

Alongside organic semiconductors, hybrid organic–inorganic materials (*e.g.*, perovskites) are popular candidates for photodetection, owing to their remarkable charge carrier mobilities and high optical absorption. Further, perovskites have large dielectric constants (*ε*_r_ ∼ 20–50), which circumvents the need for heterojunctions to efficiently generate free charges.^20^

Building on this large body of information for non-chiral OPD materials, there have been increasing efforts to develop chiral materials suitable for use in CP OPDs. These include chiral small molecules, polymers, metal-organic frameworks, and perovskites. Such materials allow for the selective absorption of one sign of CP light over the other. Furthermore, other phenomena, such as spin-polarisation in organic and hybrid semiconductors, can be manipulated as a function of their molecular chirality.^21^ For thin films of chiral materials, the absorption sensitivity to CP light is quantified by the absorbance dissymmetry or *g*-factor:^16^1
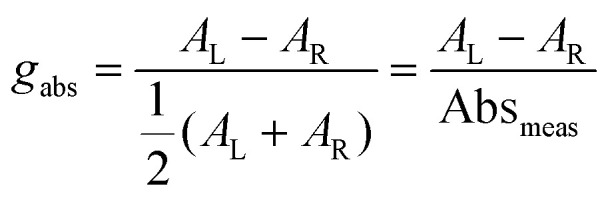
Here *A*_L_ and *A*_R_ describe the absorbance of left-handed (LH) and right-handed (RH) light. It should be noted that the measured absorption expression does not account for reflection losses occurring at the front and back surfaces of thin films. It is possible to mitigate for these reflection losses by calculating the corrected absorbance of the photoactive layer of thickness *d*:^22^2
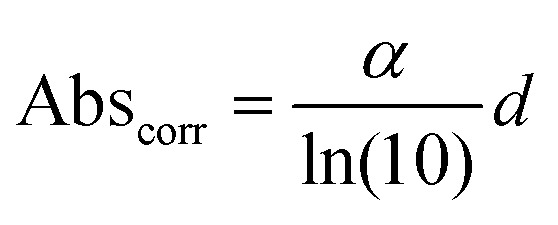
Here *α* can be determined by measuring the apparent absorbance (Abs_meas_) as a function of film thickness (*d*) and calculated using the following expression:3
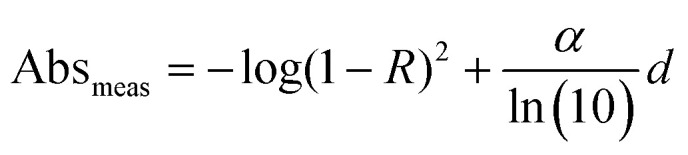
where the term in (1 − *R*)^2^ accounts for reflection losses of a free-standing film. This allows a corrected absorbance *g*-factor (*g*_corr_) to be determined:4
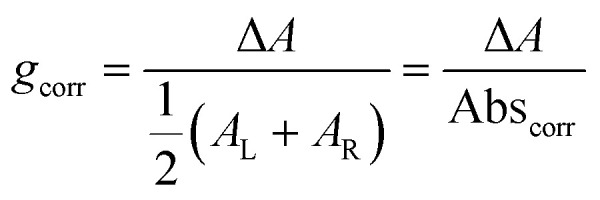
Akin to *g*_abs_ (eqn (1)), the photocurrent dissymmetry (*g*_ph_) within a CP photodetecting device describes the differential photocurrent (*I*_ph_) generated in CP illumination:5
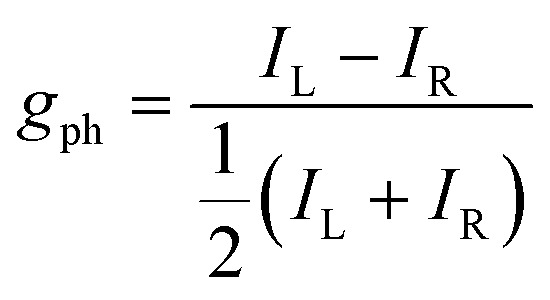
Here *I*_L_ and *I*_R_ are the photocurrents *I*_ph_ (or external quantum efficiencies, EQE, or responsivities, *R*) under LH and RH light. Note that the analogous definitions of responsivity dissymmetry (*g*_R_) and external quantum efficiency dissymmetry (*g*_EQE_) used in literature are mathematically identical to *g*_ph_ for the same intensity and wavelength of incident light, and as such will not be defined here (see ESI‡).

From a molecular perspective, *g*_abs_ depends on the strength and relative orientation of the electric (***µ***) and magnetic (***m***) transition dipole moments.^15^ For most molecular systems, |***m***| ≪ |***µ***|, which results in vanishingly small dissymmetry factors (*g*_abs_ ∼ 10^−4^–10^−3^). Strategies have emerged to increase *g*_abs_, including rational molecular design, the formation of supramolecular chiral structures in which considerable *g*_abs_ can be achieved through circular-selective scattering, and coupling between excited states on nearby chromophores.^23–29^ For example, in the solid-state, systems based on achiral π-conjugated polymers blended with chiral small molecule additives can give rise to very large *g*_abs_ (>0.1).^30^ However, these approaches can come at a cost: the twisted molecular structures required in these approaches can compromise molecular packing and impede charge transport.

## 3. Device operating principles

**Fig. 2:**
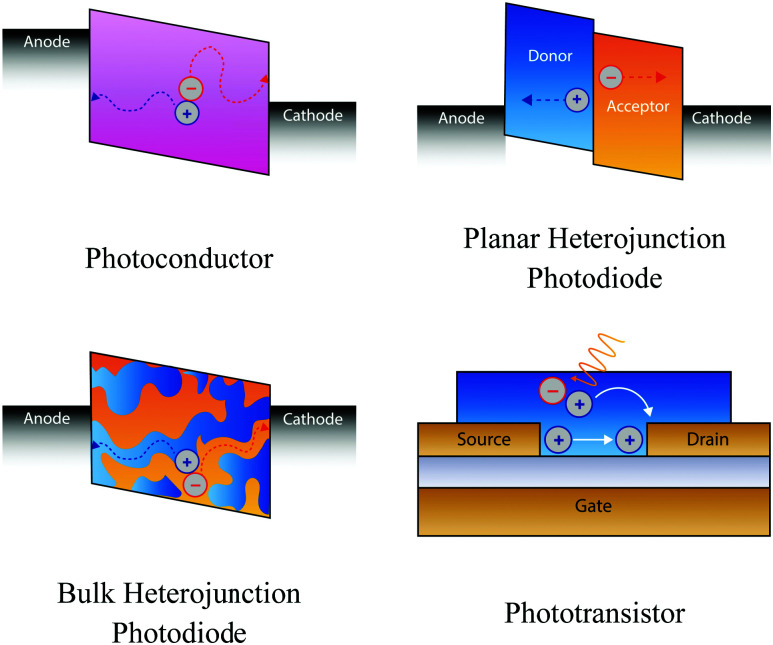
The most popular device architectures of circularly polarised photodetectors. Plasmonic devices are not discussed in this perspective.

The photophysical mechanisms that underpin photodetection depend on the device architecture. Here we introduce the most common device architectures (Fig. 2), discuss the fundamental processes at play, and highlight why certain chiral materials are particularly well suited for different architectures.

### Photoconductors

**Fig. 3:**
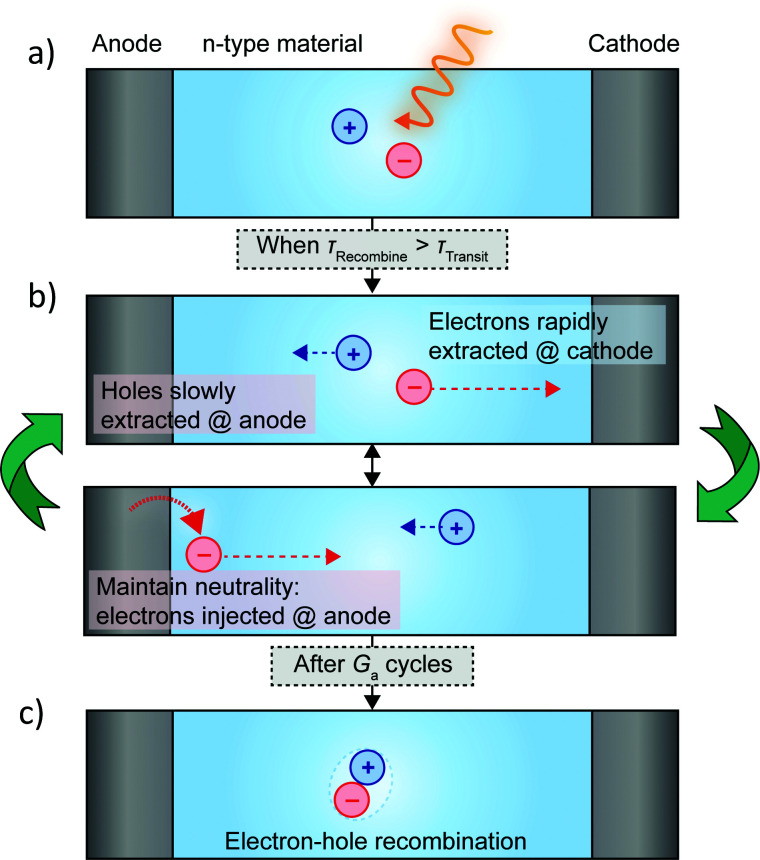
Step-by-step description of the photoconductive gain mechanism. (a) Electron and holes are excited by incident light. (b) Photogenerated electrons are rapidly extracted from the device at the positive terminal, whereas holes migrate with much slower velocity to the negative terminal. To maintain charge neutrality, electrons must be injected at the negative terminal as they are extracted from the positive terminal. This process repeats for many cycles (*G*_a_) until electron-hole recombination occurs (c).

Photoconductors are two terminal devices in which the conductivity of the active layer increases upon illumination. Optical excitation results in the generation of charge carriers and flow of a photocurrent (*I*_ph_), which is given by:6
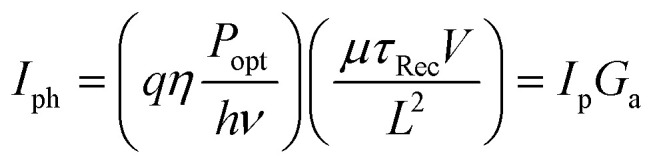
where *q* is the electron charge, *η* is the photocurrent external quantum efficiency, *P*_opt_ is the incident optical power, *hν* is the incident photon energy, *μ* is the majority carrier mobility, *τ*_Rec_ is the electron–hole recombination time, *V* is the applied voltage between the terminals, and *L* is the photoconductor electrode spacing.^11,31^*I*_p_ is the primary photocurrent (*i.e.*, *I*_ph_ in the absence of gain mechanisms) and *G*_a_ is the device gain, given by:7
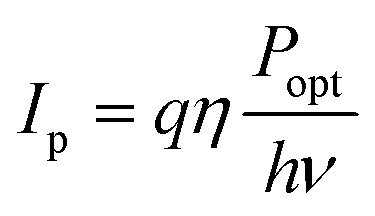
and8
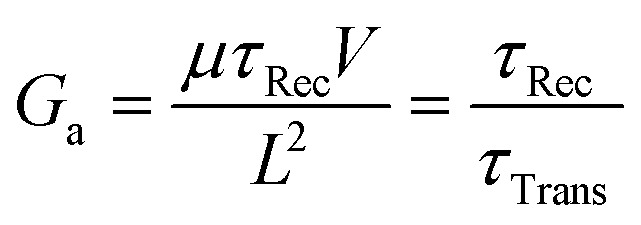
respectively, where:9
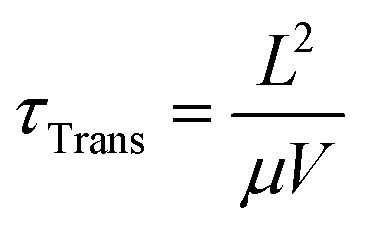
Gain arises when the electron–hole recombination time (*τ*_Rec_) is longer than the transit time for carriers to move between the two electrodes (*τ*_Trans_).^11,31^ Small *τ*_Trans_ requires small electrode spacing, *L*, which can reduce the photoactive area of the photoconductor, unless the device width is increased to compensate. In this scenario, photogenerated carriers can traverse the active layer for several cycles before recombining, with the average number of cycles equivalent to *G*_a_ (Fig. 3). This enables the ratio of electrons extracted as photocurrent and incident photons (*i.e.*, EQE) to exceed 100%.

**Fig. 4:**
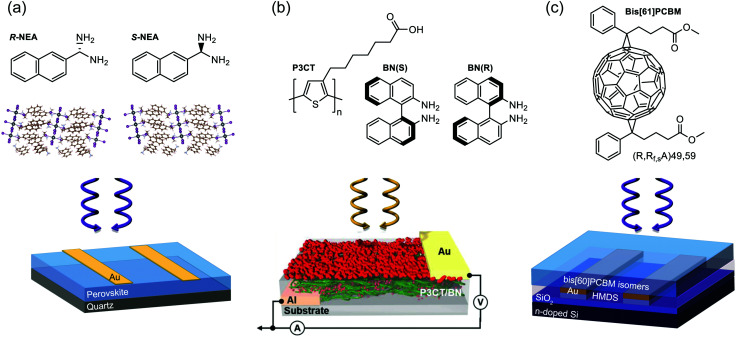
Structure of chiral active layer (top) and device architecture (bottom) for three CP selective photodetector case studies. (a) A photoconductor based on a quasi-2D perovskite using chiral 1-(2-naphthyl)ethylamine) as its organic cation.^37^ (b) A photodiode based on an achiral polymer (P3CT) and chiral dopant (BN) blend.^43^ (c) A phototransistor making use of enantiomers of bis-PCBM as its active layer.^49^

Perovskites are particularly well suited to the photoconductor geometry due to their large effective carrier mobilities (0.1–10 cm^2^ V^−1^ s^−1^) and long carrier lifetimes (10^−6^ s), which enables significant *G*_a_.^20,32–34^ Carriers with long *τ*_Rec_ persist once the light is switched off, which means that these devices can be slow to respond and have low bandwidths. Chiral perovskite photoconductors generally suffer from poor CP selectivity in absorption (*g*_abs_) owing to weak intrinsic chiroptical activity, however a number of recent examples have demonstrated that reducing the dimensionality of the perovskite structure (from quasi-2D to 2D or from 2D to 1D) can enhance *g*_abs_ by introducing greater asymmetry and helicity into the perovskite crystal structure.^35–37^ Even perovskites which demonstrate poor *g*_abs_ can generate considerable *g*_ph_, for example naphthylethylamine based quasi-2D perovskite (*g*_abs_ = 5.0 × 10^−3^, *g*_ph_ = 0.15, see Fig. 4a).^37^ A number of mechanisms have been proposed for this enhancement, such as the direct excitation of spin-polarised electrons by CPL in perovskites and the spin-selective transport of these carriers in the perovskite due to large Rashba splitting.^38–40^

### Photodiodes

The most common type of photodetector is a photodiode, which can be operated at short circuit, for low noise, low dark current detection, or under reverse bias to enhance the response speed and responsivity of the detector. The photocurrent generated in heterojunction photodiodes is limited by the exciton diffusion length of the active layer, which is typically low in organic semiconductors (∼10 nm). As such, a compromise exists between strong light absorption and efficient exciton harvesting. This has resulted in the development of various photodiode architectures, including bilayer devices, interpenetrating donor-acceptor networks (bulk heterojunctions), and tandem devices.^41^ Note that, excluding gain mechanisms such as photon downconversion, the EQE of photodiodes is restricted to 100%.^42^

In 2019 Kim *et al* demonstrated heterojunction CP OPDs with photocurrent dissymmetry (*g*_Ph_) ∼ 0.1 by blending achiral polymer, P3CT, with a chiral small molecule additive, 1,1′-binaphthyl (see Fig. 1 and 4b).^43^ Appropriate thin film processing caused the chiral small molecule to phase separate from the blend, and chirality transfer from the chiral additive to the polymer–molecule hetero-aggregates gave rise to strong CP absorption: a self-organised, circularly selective heterojunction. We have recently shown that highly selective photodiodes with *g*_Ph_ ∼ 0.9 can be realised in a simple, bilayer architecture. In these devices, selectivity to CP light occurs within the achiral polyfluorene-based (co-)polymer (F8T2): chiral small molecule (1-aza[6]helicene) additive donor layer, and exciton dissociation takes place at the donor–acceptor (C_60_) interface (see Fig. 1 for structures). Whilst chiral non-fullerene acceptors (NFAs) are yet to be realised, NFA-based photodiodes hold immense promise for next-generation devices, as they can harness longer wavelength radiation and have surprisingly long exciton diffusion lengths.^44,45^ The bandwidth of a CP photodiode is limited by the transit time and RC constant of the device, which represent the time taken for carriers to traverse the distance between the anode and cathode, and the characteristic time taken for capacitively stored charges to accumulate and discharge from the photodiode, respectively.^10^

### Phototransistors

Phototransistors are three-electrode (Source, Gate and Drain) devices in which the channel conductivity and charge injection barrier are modulated by external illumination.^46^ Akin to photodiodes, transistors operate in photoconductive and photovoltaic mode, depending on whether the voltage applied to the gate electrode (*V*_G_) is above or below the ‘threshold’ voltage (*V*_Th_) of the device. Irradiation of p-type (*i.e.*, hole transporting) organic phototransistors results in the flow of photogenerated holes to the drain electrode and photogenerated electrons flow to the source. When *V*_G_ < *V*_Th_, phototransistors behave as photoconductors, with photocurrent given by eqn (6). Above the threshold voltage (*V*_G_ > *V*_Th_) photogenerated holes contribute to the measured *I*_ph_, whilst photogenerated electrons serve to lower the injection barrier (and |*V*_Th_|), which increases *I*_ph_ further:10
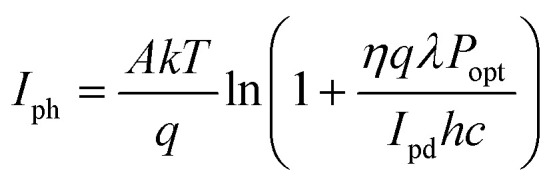
Here *A* is a proportionality constant, *kT*/*q* is the thermal voltage, *η* is the photogeneration quantum efficiency, *hc*/*λ* is the photon energy and *I*_pd_ is the dark current for minority carriers.

Both chiral small molecules (helicenes, chiral fullerenes) and chiral polymeric systems (polyfluorene-based (co-)polymers) have been assessed in CP selective phototransistors, achieving very high photocurrent dissymmetry factors (*g*_ph_ ∼ 1).^47–49^ For our own studies of phototransistors based on the chiral fullerene bis-PCBM (see Fig. 1 and 4c), we attribute the large photocurrent dissymmetry of chiral phototransistors to the greater shift of threshold voltage under the preferentially absorbed handedness of CPL.^49^ This creates a condition close to the threshold voltage of the transistor where the transistor is in the “on” state for the preferentially absorbed handedness of CPL and in the “off” state for the opposite handedness, leading to a large *g*_ph_ value of 1.3, in spite of a low *g*_abs_ of 6.3 × 10^−4^.^49^

Although phototransistors can demonstrate enhanced CP selectivity, the non-linear optical response and restricted photoactive area can bring additional complexity to evaluating their potential benefits. The development of high-mobility long recombination lifetime chiral organic semiconductors should greatly improve CP phototransistor performance.

## 4. Figures of merit

To be of any practical use CP OPDs must not only have impressive selectivity to CP light (high *g*_Ph_) but have performance metrics comparable with state-of-the-art OPDs. For example, in optical communications, a high-speed demodulation system requires a large response to the signal, a broad bandwidth to accommodate variations and minimal background noise. Here we look to establish the key parameters used to evaluate performance of OPDs and propose standard testing protocols that can ensure the accurate, non-biased evaluation of their photoresponse. We have summarised the performance metrics for CP OPDs reported so far in Table 1.

**Table 1:** Summary of the key figures of merit for all CP selective photodetectors published to date, including (from left to right) absorption dissymmetry, photocurrent dissymmetry, linear dynamic range, external quantum efficiency, responsivity, bandwidth, dark current, dark current density, detectivity, rise and fall times, on/off ratio and the wavelength at which maximum photocurrent dissymmetry occurs. The measurement conditions for these figures correspond to the same conditions which yield the greatest photocurrent dissymmetry, not simply the optimum reported value in each case. Missing figures of merit are marked by grey cells and figures of merit which do not apply to the device architecture reported are marked with an “x”. Where figures of merit have not been reported, they have been extracted from graphical data using data extraction software where possible. Note that the dissymmetry factors of photocurrent (*g*_ph_), responsivity (*g*_R_), and external quantum efficiency (*g*_EQE_) are mathematically identical for the same intensity and wavelength of incident light (see ESI), therefore, the largest of these three quantities in each study is included in the fifth column of this table. * These values have been incorrectly reported in their original articles and have been corrected here

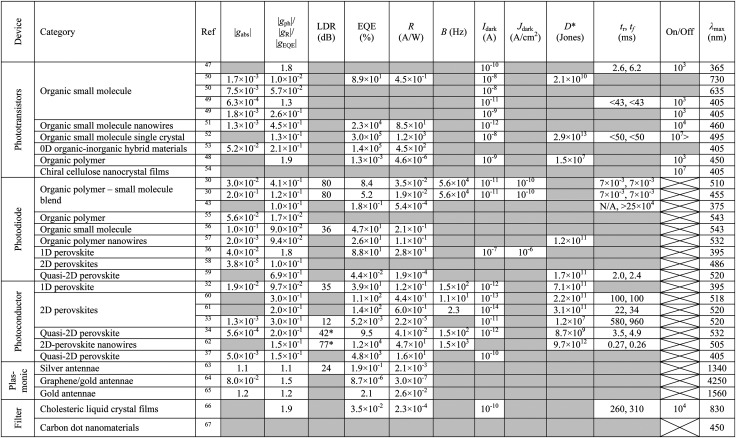

### Responsivity and external quantum efficiency

External quantum efficiency (EQE) describes the ratio of the number extracted charges to the number of incident photons at a particular wavelength, for a given applied bias. EQE is closely related to the responsivity (*R*), which quantifies the ability of a photodetector to convert light into an electrical current for an incident optical power:11
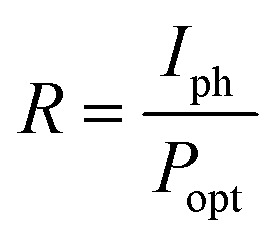
12
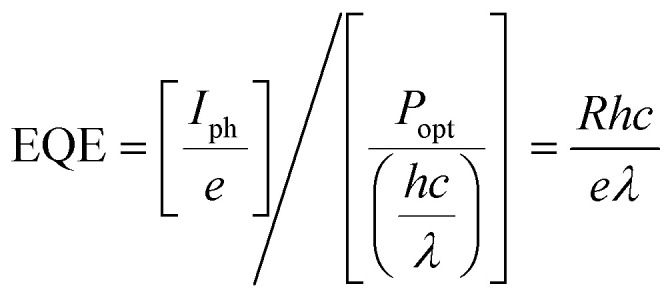
Here *P*_opt_ is the incident optical power, *e* is the elementary charge, *h* is Planck's constant, *c* is the speed of light and *λ* is the wavelength of the incident light. Whilst photoconductor gain can result in EQEs of over 100%, the EQE of photodiodes is limited to 100%, which leads to small electrical signals when exposed to weak light. Remarkably, the responsivities of 2D chiral perovskite nanowire photodetectors (*R* = 47 A W^−1^ at 505 nm) have already exceeded the responsivities of silicon-based devices (*R* = 0.6 A W^−1^ at 930 nm).^62,68^

### Linear dynamic range

The dynamic range defines the light intensity range in which the photodetector can operate. The range over which the photoresponse scales linearly with incident light intensity is referred to as the LDR:13
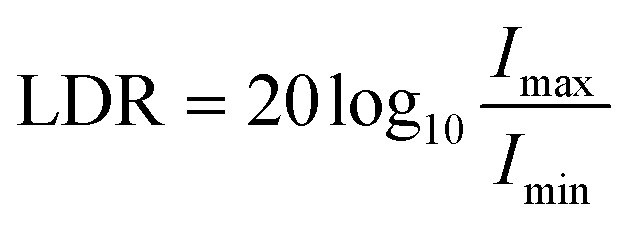
where *I*_max_ and *I*_min_ are the maximum and minimum *I*_ph_ values over which the device response is linear with increasing incident light intensity.^10^

Although an important figure of merit for OPDs, LDR is rarely reported in CP OPD literature (see Table 1). Large LDR can be achieved by suppressing noise current (reducing *I*_min_) and is desirable for machine vision applications where images may contain both intense bright spots and low light regions which must be accurately measured. Our achiral polyfluorene-based (co-)polymer: chiral small molecule (1-aza[6]helicene) photodiodes achieve an LDR of ∼80 decibels, which is considerably higher than that of InGaAs (∼66 dB) and rivals both silicon (120 dB) and non-chiral OPDs (∼100 dB).^30,69^

### Response speed

The response speed of a photodetector quantifies the time taken for the photocurrent of a photodetector to change in response to variations in incident light intensity. This establishes a limit on the speed of optical phenomena which can be detected by a given photodetector and the rate of data transfer in an optical communication system. Response speed can be quantified by measuring the bandwidth (*B*) or the rise and fall times (*t*_r_ and *t*_f_, respectively) of the photodetector.

**Fig. 5:**
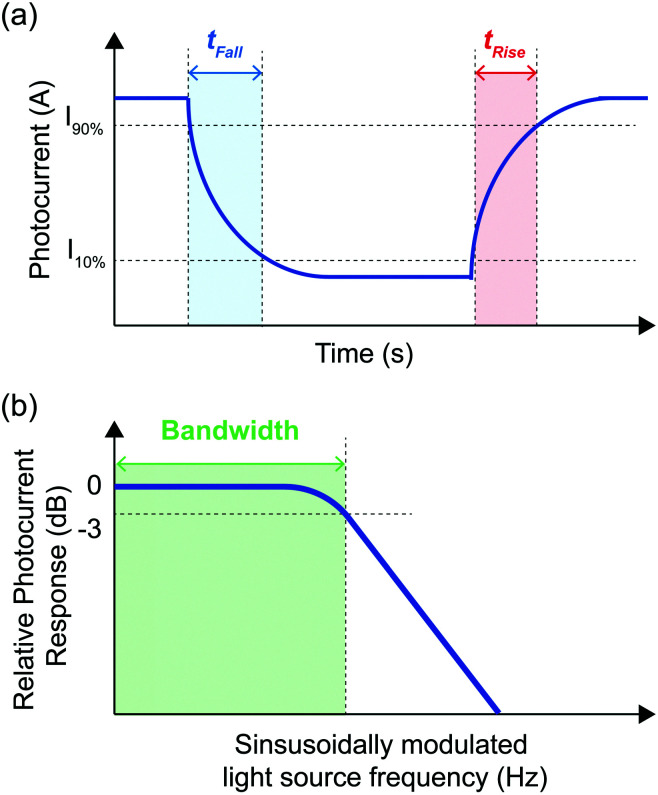
Definitions of the rise and fall times (a), and bandwidth (b) of a photodetector. (a) Rise (fall) time is the time taken for the device photocurrent to increase (decrease) from 10% (90%) to 90% (10%) of its maximum photocurrent under square wave – modulated illumination. (b) Bandwidth is the frequency of a sinusoid-modulated light source at which the photocurrent of the photodetector falls to −3 dB below the photocurrent measured at a modulation frequency of 0 Hz (*i.e.*, Continuous illumination).

The bandwidth (Fig. 5) measures how quickly a photodetector responds to light and is determined by the carrier transit time and the characteristic RC time of the device. Carrier transit time is determined by the length of the path that carriers must take to be extracted from a photodetector active layer and the strength of the electric field applied to increase the extraction rate. Parameters controlling the RC time of the device are device specific, however, any change to the device structure which reduces its total series resistance (*e.g.* reducing the distance between electrodes, reducing device contact resistance, the use of a higher mobility active layer and the use of lower sheet resistance contacts) or reduces the capacitance of the device (*e.g.* reducing the electrode area and reducing the photoactive area of the device) will reduce the characteristic RC time. In general, devices that exploit photoconductive gain have slower response times, which limits data transfer rates in optical communication systems. Whilst video applications require *B* > 10 kHz, frequencies from 100 GHz to THz are particularly promising for next-generation communications because of unused bands of the spectrum, and are essential for technological applications such as the Internet of Things (IoT), autonomous driving, and augmented reality.^70^

The rise time (*t*_r_) is the time taken for the device response to rise from 10% to 90% of its maximum value and the fall time (*t*_f_) is the time taken for the device response to fall from 90% to 10% of its maximum value (see Fig. 5).^10,36^ In dynamic imaging applications, a fast response time is essential to eliminate lag and enable fast imaging. To reliably measure pulsed signals without distortion, photodetectors require response times that are considerably faster than the pulse width to be measured. The response times depend how efficiently the photodetectors extract photogenerated charge carriers and can be improved through the judicious choice of electrodes and charge transport layers.

To date, the fastest organic CP selective photodetectors are our own photodiodes based on chiral small molecule (1-aza[6]helicene) – achiral polymer (F8T2) blends, with a bandwidth of 56 kHz (see Fig. 1).^30^ While suitable for imaging applications, which require frame rates of approximately 100 Hz, this bandwidth falls short of the bandwidth of InGaAs detectors used in fibre optic communications, with bandwidths in the 10 GHz range and rise times of the order of picoseconds.^10,71^ The low trap density and high charge carrier mobility of hybrid perovskite materials afford them both high LDR and ultrafast response times. Recently, response times of <10 ns have been reported in non-chiral perovskite/organic bulk heterojunction photodetectors based on MAPbI_3_ and F8IC (see Fig. 1).^72^ The fast response time is attributed to (i) the high hole mobility of MAPbI_3_, (ii) the high electron mobility of F8IC and (iii) the suppression of RC delay times due to the reduced active area of these devices (and therefore reduced device capacitance, *C*). The use of chiral active layers with high charge carrier mobilities and devices with small active areas should be further explored to increase the speed of CP selective detectors to be compatible with communication applications.

### On–off ratio

On–off ratio *R*_on/off_ defines the ratio between the device drain current for the device in the on- and off-state for phototransistors:14
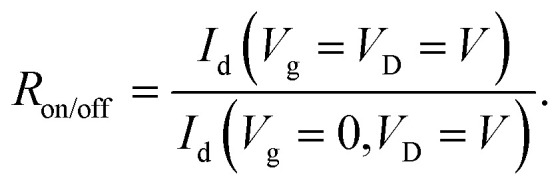
A high *R*_on/off_ value is desirable as this indicates that a low driving voltage is required to achieve a good signal-to-noise ratio.^17^ This reduces the energy consumption of circuits based on organic transistors and enables their use in low power applications such as IoT devices.

To date, the highest recorded on–off ratio for a CP selective phototransistor is over 10^7^, exceeding the on–off ratios of some inorganic phototransistors.^52,73^

### Dark current and noise current

The dark current describes the flow of charge carriers through a photodetector in the dark, whilst the noise current describes random fluctuations in the dark current. Several mechanisms can generate the noise current inside organic photodetectors, which have been summarised by Hirsch *et al.*^74^ The minimum light power that can be detected is quantified by the noise equivalent power (NEP):15
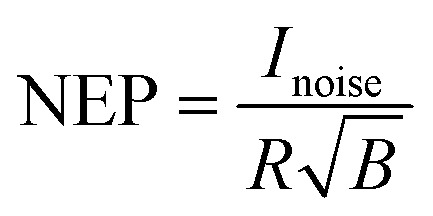
where *B* is the electrical bandwidth. The specific detectivity (D*) is the inverse of NEP normalised by the square root of the active area, and can be used to compare the detectivity of devices with different active areas:16
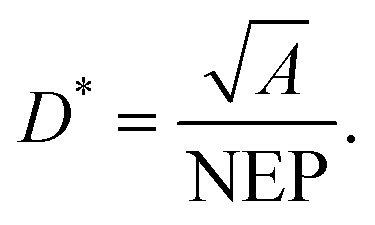
The pursuit of highly sensitive photodetectors with large signal-to-noise ratios that do not require cooling has resulted in considerable effort to suppress the dark and noise currents and enhance D*, yet a comprehensive understanding of the intrinsic and external sources is currently lacking.

The largest detectivity for a CP selective photodetector was reported as 2.9 × 10^13^ Jones for a phototransistor based on an organic small molecule single crystal, relative to a value of 2.3 × 10^11^ Jones for a typical Si photodiode.^52,75^

### Operational lifetime

The operational lifetime of a photodetector represents time for which the photoconductor responsivity remains approximately constant under fixed bias and illumination conditions. For CP selective photodetectors, this must also include the stability of the dissymmetry of photocurrent over time.

Photodetectors typically undergo an initial rapid degradation in device responsivity, referred to as “burn-in”, followed by a slower, more stable decay period (post-burn-in), which must be accounted for in the measurement of operational lifetime.^76,77^ This is particularly common in perovskite devices.^77^

To the best knowledge of the authors, only one report of the operational lifetime of a CP selective photodetector has been published. This device was a quasi-2D perovskite photoconductor with a reported operational lifetime of one month.^37^

## 5. Measurement protocols

The fast-paced interdisciplinary nature of photodetector research has resulted in incomplete reporting of figures of merit (see Table 1), which limits advances in molecular and device design. Whilst separate papers outline the experimental procedures required to accurately characterise device performance of non-chiral OPDs^78^ and, separately, the chiroptical activity of chiral materials,^79^ here we summarise our own best practice for the complete characterisation of CP OPDs.

### Circular dichroism spectroscopy

The circular dichroism (CD) and *g*_abs_ of a solution or film are measured using a CD spectrometer. For solid state samples, CD spectra should be collected with the sample in various orientations (face-on, back-on, at 0° and rotated at 90° around the optical axis). This allows for the identification of artefacts due to linear dichroism and linear birefringence, which would appear as a shift or inversion of the CD spectrum on rotation or flipping the side of the sample facing the light source.^79^ Linear artefacts may also be identified through the use of Mueller Matrix Spectroscopic Ellipsometry and Mueller Matrix Polarimetry Imaging, which are capable of resolving the circular dichroism, circular birefringence, linear dichroism and linear birefringence spectra for a sample simultaneously.^79,80^

### Responsivity and EQE

**Fig. 6:**
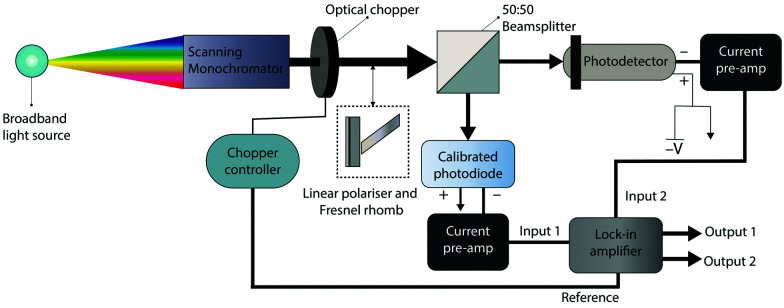
Experimental setup for the measurement of EQE and responsivity. The device under test is illuminated with monochromatic light from a white light source which has passed through a scanning monochromator. Monochromatic light is chopped by an optical chopper and the chopped light is split equally between a calibrated photodiode and the device under test using a beamsplitter. A linear polariser and Fresnel rhomb can be added in front of the beam to produce a circularly polarised beam. The photocurrent from the device under test and the calibrated photodiode are converted to voltages, which are measured *via* sensitive lock-in detection using a lock-in amplifier, using the chopper controller signal as a reference frequency.

Responsivity and EQE are measured using the equipment shown in Fig. 6. Chopped monochromatic light is used to generate a time-varying photocurrent in both the device under test and a calibrated photodiode using beamsplitter cube. The photocurrents of both the device under test and the calibrated photodiodes are converted to voltages using current preamplifiers. Together with a reference signal from the optical chopper, these two voltage signals are inputted into a lock-in amplifier. The voltages measured by the lock-in are used to calculate the photocurrent of the device under test, and calibrated photodiode using the current-to-voltage gain of the current preamplifiers. The photocurrent of the calibrated photodiode is used to determine the total incident optical power and is used with the photocurrent of the device under test in eqn (11) and (12) to determine the device EQE and responsivity. A chopped light source and lock-in amplifier considerably reduces the noise of the measured photocurrent, which enables measurements of EQE with superior accuracy (<±0.1%).

By placing a linear polariser and Fresnel rhomb (a broadband quarter-waveplate) in the path of the chopped monochromatic beam, EQE and responsivity can be measured under circularly polarised light in the wavelength range of 400–1550 nm.^81^ The two EQE spectra of the device under L-CPL and R-CPL can then be used to calculate a photocurrent dissymmetry spectrum.

### Linear dynamic range

**Fig. 7:**
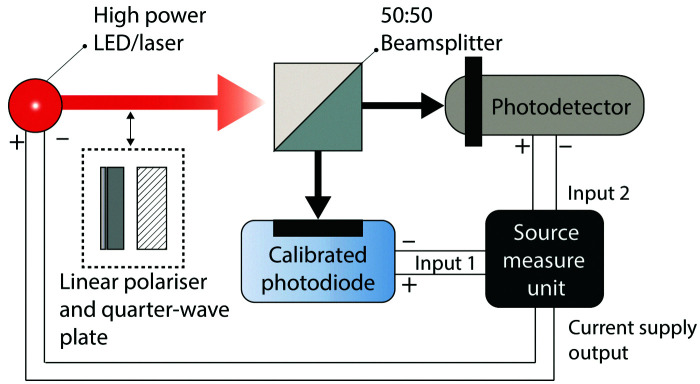
Experimental setup for measuring the linear dynamic range. Light from a high power LED or laser is split equally between a calibrated photodiode (for measuring light intensity) and the device under test using a beamsplitter. A linear polariser and quarter-wave plate may be added in the path of the light source for a circularly polarised beam. The intensity of the light source is controlled using a digital current source and the photocurrent of the calibrated photodiode and device under test are measured using a source-measure unit.

The LDR of a photodetector is measured using the setup shown in Fig. 7. A high-power laser or monochromatic LED is used to illuminate the device under test and the intensity of the incident light is monitored using a calibrated photodiode. The intensity of the light source is gradually increased from the off state while the photocurrent of the device under test and the intensity of the incident light (*via* the calibrated photodiode photocurrent) are monitored using the source measure unit. The intensity of the incident light is steadily increased until the device under test deviates from a linear relationship between photocurrent and incident light intensity. The minimum and maximum photocurrents in this linear region are then applied to eqn (13) to determine the LDR.

### Response speed

**Fig. 8:**
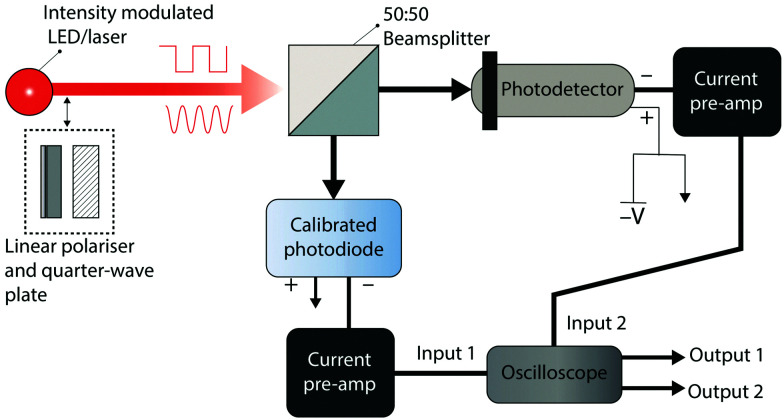
Experimental setup for measuring the bandwidth, rise time, fall time, and operational lifetime. Light from a high-power LED or laser is split equally between a calibrated photodiode (for measuring light intensity) and the device under test using a beamsplitter. A linear polariser and quarter-wave plate may be added in the path of the light source for a circularly polarised beam. For operational lifetime, rise time and fall time measurements, a square wave modulated light source should be used and for bandwidth measurements, a frequency tuneable sinusoidally modulated light source should be used. The photocurrent of the device under test and the calibrated photodiode are converted to voltages using current preamplifiers and monitored using an oscilloscope.

Detector *B* is measured using the setup shown in Fig. 8. The device under test is illuminated with a frequency tuneable sinusoidally modulated light source. The current output of the photodetector is converted to a voltage using a current preamplifier and the peak-to-peak voltage of the photodetector is monitored using an oscilloscope. The frequency (*f*) of the light source is gradually increased until the peak-to-peak voltage of the photodetector signal (*V*_sin_(*f*)) satisfies the 3 dB condition:17
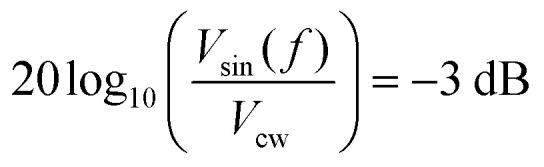
where *V*_cw_ is the continuous wave response of the photodetector to an unmodulated light source with an intensity equal to the peak intensity of the sinusoidally varying light source. This is the “Bandwidth” or “3 dB cut-off frequency” of the device.

The *t*_r_ and *t*_f_ of a photodetector can be extracted using the same setup which is used for the measurement of *B*. The photodetector is illuminated using a square wave modulated light source with a tuneable switching frequency. The frequency of modulation should be selected so that the device under test produces an oscilloscope signal of the form shown in Fig. 5a, with the device reaching an equilibrium voltage when the light source is in both the on and off states. Most oscilloscopes have built-in functions for extracting the average *t*_r_ and *t*_f_ once a signal of this form is acquired. *t*_r_ and *t*_f_ should not be extracted using the current measurement of a digital source measure unit, as the measurement sampling frequency is often inadequate for accurate measurements of response times below 10 ms.

In the measurement of *B*, *t*_r_ and *t*_f_, it is essential that the light source, modulation source, current preamplifier and oscilloscope have electrical bandwidths greater than the bandwidth of the photodetector under test, otherwise the lowest bandwidth component of the testing apparatus will be measured instead of the true bandwidth or response times of the device. For example, if the bandwidth of a photodiode with a −3 dB cut-off frequency of 100 kHz is measured using a current preamplifier with a bandwidth of 10 kHz, an incorrect bandwidth of approximately 10 kHz or less will be measured.

### Operational lifetime

The operational lifetimes of both CP and non-CP photodetectors have been measured using several different methods in the literature. To standardise this measurement, we suggest a similar approach to that taken by Kielar *et al.* and Wang *et al.*, defining the operational lifetime as the time required for the responsivity or photocurrent dissymmetry factor of a device to fall to 80% of their post-burn-in values, whichever comes first.^76,77^

To measure responsivity and *g*_ph_ as a function of device operation time, we propose that the operational lifetime of a device should be measured under pulsed CP illumination, alternating between L-CP illumination, dark conditions, R-CP illumination, and dark conditions repeatedly. This can be achieved using the setup shown in Fig. 8. To avoid artificially inflating the device operational lifetime, device testing conditions (intensity, wavelength, and photodetector bias) should match those conditions required for the maximum photocurrent dissymmetry reported in the study.

### Dark current and noise current

The *I*_dark_ of a photodetector is measured as simply the current flow through the photodetector under dark conditions.

The NEP of a photodetector can be determined by measuring the voltage output of a current preamplifier connected to the device under test in dark conditions using a lock-in amplifier. Most modern digital lock-in amplifiers can provide the noise spectral density of the input signal which, when divided by the responsivity of the detector at a given wavelength, yields the noise equivalent power at that wavelength. This can, in turn, be used to calculate the device detectivity using eqn (16).

## 6. Conclusions and outlook

Circularly polarised organic photodetectors will facilitate future technologies based on image detection, chemical sensing, encrypted communications, and environmental monitoring. The diversity of accessible chiral functional materials that absorb circularly polarised light introduces a wide range of new possibilities, which has inspired considerable research interest in both academia and industry. Whilst reported devices often show impressive chiroptical responses, their device performances are often inferior when compared to non-chiral photodetectors. Further, complete and rigorous characterisation of the critical photodetector parameters (*e.g.*, linear dynamic range, bandwidth, detectivity) is rarely provided, which limits their application in real-world devices. In this tutorial review we have summarised the most important figures of merit for circularly polarised organic photodetectors and provided detailed descriptions of how to measure them.

Innovations in molecular design, novel device architectures and stronger collaborations between materials chemists and device physicists will advance the field. Optical sensing from the ultraviolet to the infrared is essential for a variety of industrial and scientific applications. To this end, chiral functional materials with photoresponses that span the full range from the UV to the NIR will likely show promise. Chiral materials with outstanding electronic properties (*e.g.*, minimal defects, a low trap density and high charge carrier mobilities) are essential for high-performance devices. Here, chiral organic–inorganic systems (*e.g.*, perovskites) and supramolecular assemblies are the most attractive candidates. Finally, exciton dissociation and the efficient extraction of charge carriers will require the use of multi-layer devices and optimised heterojunction structures with intermixed donor and acceptor domains.

We hope that this review will help to advance this exciting and growing field and inform the design of novel materials/devices with outstanding chiroptical properties and device performance.

## Conflicts of interest

There are no conflicts to declare.

## Supplementary Material

TC-010-D2TC01224C-s001
