# Correction: Best practices in the measurement of circularly polarised photodetectors

**DOI:** 10.1039/d2tc90220f

**Published:** 2022-11-07

**Authors:** Matthew D. Ward, Wenda Shi, Nicola Gasparini, Jenny Nelson, Jessica Wade, Matthew J. Fuchter

**Affiliations:** Department of Physics, Imperial College London South Kensington Campus London SW7 2AZ UK; Centre for Processable Electronics, Imperial College London South Kensington Campus London SW7 2AZ UK jessica.wade@imperial.ac.uk m.fuchter@imperial.ac.uk; Department of Chemistry and Molecular Sciences Research Hub, Imperial College London White City Campus, 82 Wood Lane London W12 0BZ UK; Department of Materials, Imperial College London South Kensington Campus London SW7 2AZ UK

## Abstract

Correction for ‘Best practices in the measurement of circularly polarised photodetectors’ by Matthew D. Ward *et al.*, *J. Mater. Chem. C*, 2022, **10**, 10452–10463, https://doi.org/10.1039/D2TC01224C.

The authors regret errors which appeared in Fig. 4 of the published article, where the structures of *R*- and *S*-NEA in Fig. 4a were incorrectly shown containing two NH_2_ groups, instead of one NH_2_ group and one CH_3_ group. The corrected version of Fig. 4 is shown below:
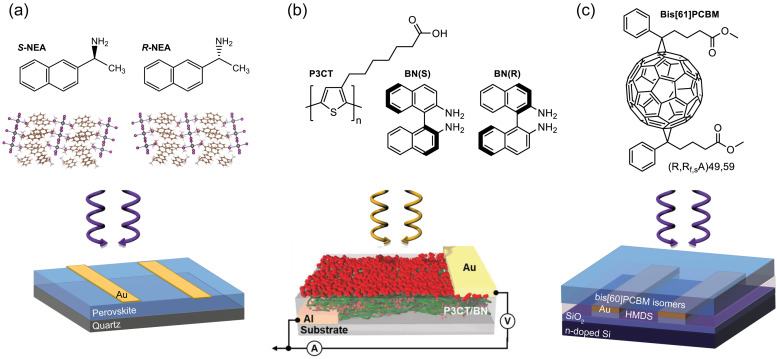


The Royal Society of Chemistry apologises for these errors and any consequent inconvenience to authors and readers.

## Supplementary Material

