# Supplementary material for: Best practices in the measurement of circularly polarised photodetectors
Source: J Mater Chem C Mater. 2022 Jun 30;10(29):10452–63. doi: 10.1039/d2tc01224c (PMC9332130; doi:10.1039/d2tc01224c)
Supplement: TC-010-D2TC01224C-s001 [file TC-010-D2TC01224C-s001.pdf]

Electronic Supplementary Material (ESI) for Journal of Materials Chemistry C.
This journal is © The Royal Society of Chemistry 2022

Best practices in the measurement of circularly polarised photodetectors

Matthew D. Ward,[a,b] Wenda Shi,[c,b] Nicola Gasparini,[c,b] Jenny Nelson,[a,b] Jessica Wade,[d,b] Matthew J. Fuchter[c,b]**

Dedication: To the memory of Professor Alasdair James Campbell.

[a] Department of Physics, Imperial College London, South Kensington Campus, London SW7 2AZ, United Kingdom. [b] Centre for Processable Electronics, Imperial College London, South Kensington Campus, London SW7 2AZ, United Kingdom. [c] Department of Chemistry and Molecular Sciences Research Hub, Imperial College London, White City Campus, 82 Wood Lane, London W12 0BZ, United Kingdom. [d] Department of Materials, Imperial College London, South Kensington Campus, London SW7 2AZ, United Kingdom.

SUPPLEMENTARY INFORMATION

Equivalence of Photocurrent Dissymmetry, Responsivity Dissymmetry and External Quantum Efficiency Dissymmetry

The expression for the dissymmetry factor of photocurrent is given by

$$g_{ph} = \frac{I_L - I_R}{\frac{1}{2}(I_L + I_R)}$$

Where I_L and I_R are the photocurrents of the photodetector under left- (L-) and right-handed circularly polarised light (R-CPL) respectively. Similarly, the dissymmetry factors of responsivity, R, and the external quantum efficiency, EQE, are given by:

$$g_R = \frac{R_L - R_R}{\frac{1}{2}(R_L + R_R)}$$

$$g_{EQE} = \frac{EQE_L - EQE_R}{\frac{1}{2}(EQE_L + EQE_R)}$$

where R and EQE represents the responsivity and EQE of the photodetector and subscripts L and R indicate left- and right-handed circularly polarised illumination, respectively. EQE and responsivity are related by the following expression:

$$EQE = \frac{Rhc}{\lambda e}$$

Substituting this into the expression for g_{EQE}:

$$g_{EQE} = \frac{\left[\frac{R_L hc}{\lambda e}\right] - \left[\frac{R_R hc}{\lambda e}\right]}{\frac{1}{2}\left(\left[\frac{R_L hc}{\lambda e}\right] + \left[\frac{R_R hc}{\lambda e}\right]\right)} = \frac{R_L - R_R}{\frac{1}{2}(R_L + R_R)} = g_R$$

demonstrating that g_{EQE} is identical to g_R when measured under the same experimental conditions. Further, R may be expressed as:

$$R = \frac{I_{ph}}{P_{opt}}$$

Where I_{ph} is the measured photocurrent and P_{opt} is the total optical power incident on the active area of the device. Applying this to the expression for g_R yields:

$$g_R = \frac{\left[\frac{I_{ph}}{P_{opt}}\right]_L - \left[\frac{I_{ph}}{P_{opt}}\right]_R}{\frac{1}{2}\left(\left[\frac{I_{ph}}{P_{opt}}\right]_L + \left[\frac{I_{ph}}{P_{opt}}\right]_R\right)}$$

When the measurement of dissymmetry factor of responsivity is carried out correctly, P_{opt} under L-CPL and R-CPL is the same, thus

$$g_R = \frac{\left[\frac{I_L}{P_{opt}}\right] - \left[\frac{I_R}{P_{opt}}\right]}{\frac{1}{2}\left(\left[\frac{I_L}{P_{opt}}\right] + \left[\frac{I_R}{P_{opt}}\right]\right)} = \frac{I_L - I_R}{\frac{1}{2}(I_L + I_R)} = g_{ph}$$

Where we have used the following straightforward relationships: $[I_{ph}]_L = I_L$, $[I_{ph}]_R = I_R$ and $[P_{opt}]_L = [P_{opt}]_R = P_{opt}$.

In summary, we have shown that

$$g_{EQE} = g_R = g_{ph}$$

for the same intensity and wavelength of incident light and thus, these figures of merit are equivalent and do not need to be individually defined or measured for a defined set of experimental conditions. Differences in the measurement of these three quantities can only arise from different measurement conditions, such as wavelength of excitation, applied bias (including gate biases for photoFETs), intensity of illumination and device dimensions, to name the most obvious examples.

Definition of and Generation of Left- and Right-handed Circularly Polarised Light

Circularly polarised light is a polarisation of light in which the electric field vector rotates in a circle as it propagates through space. Circularly polarised light exists in two forms: left- (L-) and right-handed circularly polarised light (R-CPL). Two conventions for the definition of L-CPL and R-CPL exist: one that is traditionally used in the field of engineering, conforming with IEEE Standard 149™-1979[1] and a second standard which, whilst unnamed, is often referred to as the "Physics" definition. Unfortunately, these two standards define L-CPL and R-CPL oppositely (i.e., L-CPL in the IEEE standard is R-CPL in the Physics standard and R-CPL in the IEEE standard is L-CPL in the Physics standard), leading to frequent confusion between authors due to the lack of a single "correct" standard. While there is no "correct" definition of L- and R-CPL, it is essential that, within a given study, the definition of L-CPL and R-CPL is self-consistent. Specifically, measurements such as circular dichroism and photocurrent dissymmetry should ideally make use of the same polarisation standard.

Figure S1 demonstrates the definitions of L- and R-CPL used by the Applied Photophysics Chirascan™, which is used in the measurement of circular dichroism of optically active materials and has been used extensively in our previous work.[2] This instrument adopts the Physics standard definition of CPL and as such, we use this standard in our own studies to ensure internal consistency.

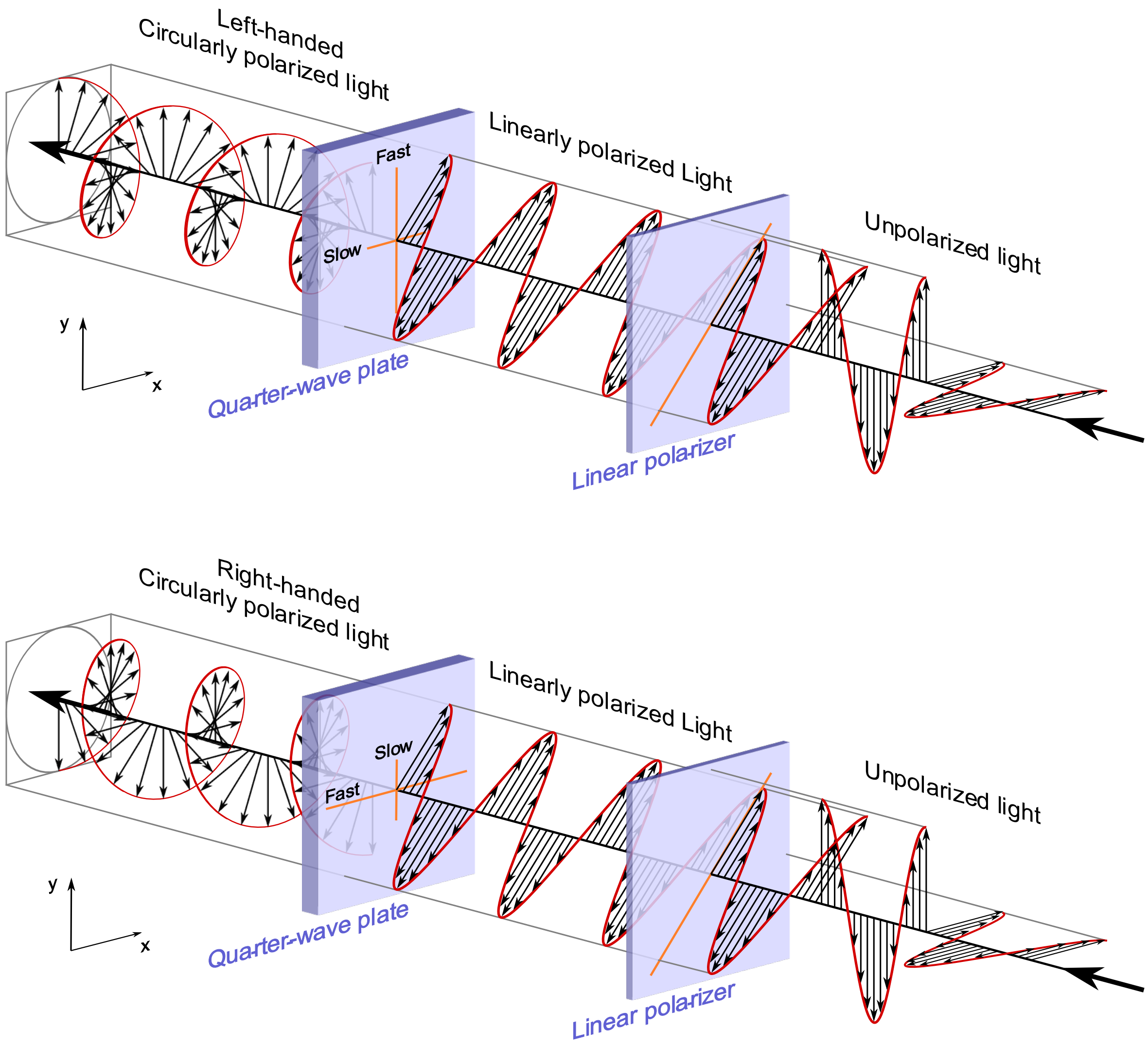

Figure S1: Definitions of left- (top) and right-handed (bottom) circularly polarised light as used in the Applied Photophysics Chirascan™.

As shown in figure S1, a linear polariser is used to align the electric field vector from an unpolarised light source in one plane at a 45° relative to the x- and y-axes of the optical coordinate system. This linearly polarised electric field can be considered to be composed of two in-phase perpendicular sinusoidal electric fields of equal magnitude, with one aligned along the x-axis and the other aligned along y-axis of the coordinate system. The linearly polarised electric field is then passed through a quarter-wave plate (QWP), which has two orthogonal axes – the fast-axis and slow axis. QWPs take many forms (e.g., birefringent crystals, Fresnel rhombs and photoelastic modulators) but all have the same effect: the electric field component aligned along the fast-axis leaves the quarter-wave plate with a phase lead of 90° (a quarter-wave) ahead of the electric field component aligned along the slow axis.

By aligning the fast-axis with the y-axis of the coordinate system, the y-component of the electric field is advanced by a quarter-wave relative to the x-component, producing L-CPL. Conversely, by aligning the fast-axis with the x-axis of the coordinate system, the x-component of the electric field is advanced by a quarter-wave relative to the y-component, producing R-CPL. Note that the QWP should be rotated when switching between L-CPL and R-CPL (as shown in Figure S1) as any slight linear polarisation in the light source will lead to changes in P_{opt} transmission through the linear polariser and QWP if the linear polariser is rotated. This leads to a violation of the assumption that $[P_{opt}]_L = [P_{opt}]_R = P_{opt}$ which is essential for the accurate calculation of several CPL parameters such as g_{ph} as discussed in section 1.

To ensure that the polarisation generated in figure 1 is a pure circular polarisation, as opposed to a linear or elliptical polarisation, it is advised that a commercial polarimeter is used to determine the polarisation ellipse of the polarised light. For a perfect circular polarisation, the shape of this ellipse should be circular.

References

1 A. Standards Committee, *IEEE Standard Test Procedures for Antennas IEEE-SA Standards Board American National Standards Institute*, 1965.

2 Understanding polarization, https://www.photophysics.com/circular-dichroism/chirascan-technology/understanding-polarization/, (accessed May 26, 2022).
